# Monitoring of circulating tumor DNA allows early detection of disease relapse in patients with operable breast cancer

**DOI:** 10.1002/1878-0261.70170

**Published:** 2025-11-27

**Authors:** Kristin Løge Aanestad, Marie Austdal, Oddmund Nordgård, Gunnar Mellgren, Satu Oltedal, Marie L. Austbø, Ylva H. Vignes, Thomas Helland, Kristin Jonsdottir, Tone H. Lende, Emilius A. M. Janssen, Bjørnar Gilje, Kjersti Tjensvoll, PBCB study group, Gunnar Mellgren, Tone Hoel Lende, Anette Heie, Kristin Viste, Anita Røyneberg Alvheim, Emiel AM Janssen, Kristin Jonsdottir, Ann Cathrine Kroksveen, Thomas Helland, Einar Gudlaugsson, Oddmund Nordgård, Satu Oltedal, Kristin Løge Aanestad, Jan Terje Kvaløy, Kirsten Lode, Kari Britt Hagen, Marie Austdal, Ylva H. Vignes, Siri Tungland Sola, Nina Egeland Amundsen, Finn Magnus Eliassen, Emeritus Ernst A Lien

**Affiliations:** ^1^ Department of Hematology and Oncology Stavanger University Hospital Norway; ^2^ Department of Research Stavanger University Hospital Norway; ^3^ European Liquid Biopsy Society (ELBS) Hamburg Germany; ^4^ Hormone Laboratory, Department of Medical Biochemistry and Pharmacology Haukeland University Hospital Bergen Norway; ^5^ Department of Clinical Science University of Bergen Norway; ^6^ Department of Pathology Stavanger University Hospital Norway; ^7^ Department of Surgery Stavanger University Hospital Norway; ^8^ Department of Chemistry Bioscience and Environmental Engineering, University of Stavanger Norway; ^9^ Institute for Biomedicine and Glycomics Griffith University Australia

**Keywords:** breast cancer, circulating tumor DNA, liquid biopsy, minimal residual disease, relapse

## Abstract

Breast cancer is known for late recurrences, yet current follow‐up lacks radiological or blood‐based monitoring for systemic relapse. This study evaluated circulating tumor DNA (ctDNA) monitoring for early detection of systemic relapse after curative treatment. In this case–control study of 70 patients with operable breast cancer (35 with relapse and 35 without relapse), blood samples were collected every 6–12 months during a median 8.3‐year follow‐up. ctDNA was analyzed by targeted DNA sequencing using Oncomine™ Breast cfDNA Research Assay v2, and results were compared to genetic analysis of tumor and metastasis biopsies. ctDNA was detected at relapse in 19 of 35 (54%) patients with disease relapse and preceded clinical or radiological relapse detection in 17, with a median lead time of 10.3 months. In 13 (68%) patients, there was concordance with tumor mutations, and in seven patients, there was also concordance with metastasis. Among the relapse‐free patients, seven were ctDNA‐positive postsurgery, and only one of them had a match among the tumor variants. These findings suggest serial ctDNA analysis may enable earlier detection of systemic relapse in patients with operable breast cancer.

## Graphical abstract

Monitoring circulating tumor DNA (ctDNA) in patients with operable breast cancer can reveal disease relapse earlier than radiology in a subset of patients. The failure to detect ctDNA in some patients with recurrent disease suggests that ctDNA could serve as a supplement to other monitoring approaches.

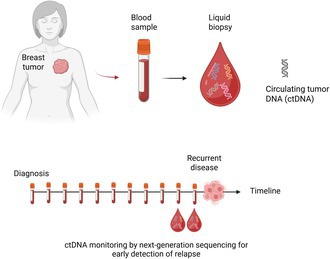

**Abbreviations**
cfDNA: circulating cell‐free DNA
CHIP: clonal hematopoiesis of indeterminate potential
CNV: copy number variations
ctDNA: circulating tumor DNA
emPCR: emulsion PCR
FFPE: formalin fixed, paraffin embedded
IHC: immunohistochemistry
INDELs: insertions and deletions
NGS: next‐generation sequencing
PBCB: prospective breast cancer biobank
SNVs: single‐nucleotide variants
TC: tumor content
VAF: variant allele frequency

## 1 Introduction

Breast cancer is the most common cancer among women worldwide, with more than 2.3 million new cases annually [1, 2]. It is also the first leading cause of cancer‐related deaths in women, annually claiming over 600 000 lives [2]. Although its incidence continues to rise, advancements in diagnostics and treatment have led to a decline in mortality rates. Across the European Union the breast cancer incidence in 2022 was 147.6 and the mortality rate was 34.1 deaths per 100.000 females [3, 4]. The five‐year relative survival was approximately 85%, while certain high‐income countries within the EU had a five‐year survival rate ranging from 90% to 93% [5].

A characteristic of breast cancer is its propensity for late relapses, which can manifest up to 30 years and beyond after primary treatment [3]. Approximately 30% of the patients with primary operable breast cancer experience disease relapse [6]. Current follow‐up of this patient group includes clinical examination and radiological imaging of the breast to detect local relapses. Systemic relapse often remains undetected until the disease has advanced significantly. In this respect, we need to develop enhanced methods to detect minimal residual disease, with the future aim of determining whether additional therapy could improve outcomes for these patients [7].

Liquid biopsies, which comprise the analysis of tumor components circulating in body fluids such as blood, have demonstrated promising clinical utility in the management of various solid tumors [8, 9, 10]. Circulating tumor DNA (ctDNA) is cell‐free tumor‐derived DNA present in the plasma fraction of peripheral blood in cancer patients [11, 12]. ctDNA may originate from apoptotic and necrotic tumor cells in the primary tumor or metastases [13]. In this respect, ctDNA shows promising biomarker properties, both as a potential surrogate marker for tumor load and by providing indirect access to the entire tumor genome [11].

In clinical medicine today, circulating cell‐free DNA is widely used, for example in noninvasive prenatal testing [14]. Moreover, in clinical oncology, the use of ctDNA is becoming increasingly relevant. In lung cancer, the examination of ctDNA has been a valuable alternative when it is difficult to get a standard biopsy. ctDNA sequencing is also used in lung cancer diagnostics to detect changes in the mutational status both to guide treatment and to follow treatment response [15].

Several studies, including multiple meta‐analyses, have highlighted the potential of using ctDNA to measure treatment response in breast cancer patients during neoadjuvant treatment, and as an additional method for more accurate stratification of the risk of relapse [16, 17, 18, 19]. The presence of ctDNA at baseline and its persistence following neoadjuvant treatment are shown to strongly correlate with an increased risk of relapse [10, 19]. Some studies also show the value of serial postoperative ctDNA assessment. These studies demonstrate that ctDNA detection after surgery is associated with a higher risk of relapse, most consistently for triple‐negative patients, but there is also a strong correlation between ctDNA detection and recurrence for the hormone receptor positive patients [7, 20]. The genomic diversity and heterogeneous mutational landscape of breast cancer have, however, important implications for the detection threshold of ctDNA. Both the specific mutations identified and the extent of ctDNA shedding can vary considerably between patients, as well as between different tumor clones within the same patient [21, 22].

Few studies have investigated longitudinal ctDNA monitoring after breast cancer surgery [7]. In the Prospective Breast Cancer Biobank (PBCB) [1] study, we have prospectively recruited patients with primary operable breast cancer and collected blood samples every 6 or 12 months for up to 11 years. In the current study, we have selected 70 of the enrolled PBCB patients in a case–control design to investigate if the ctDNA level increases at the time of disease progression, and whether a ctDNA increase can reveal systemic relapse earlier than conventional radiological imaging and routine clinical follow‐up.

## 2 Materials and methods

### 2.1 Patient samples

The patients (*n* = 70) included in this study were enrolled in the Prospective Breast Cancer Biobank (PBCB) [1], at Stavanger and Haukeland University Hospitals, during the years 2013–2016. At the time of inclusion, the patients were diagnosed with operable breast cancer, displayed no clinical evidence of metastatic disease and were considered cancer‐free after surgery and adjuvant therapy. All patients recruited in the PBCB study prospectively provided tissue samples at the time of surgery and blood samples every 6–12 months during a follow‐up period of up to 11 years. In this case–control study, we selected tissue and plasma samples obtained from 35 operable breast cancer patients experiencing systemic relapse during a median follow‐up of 8.29 years, and 35 matched operable breast cancer patients, who were relapse‐free at least 5 years after primary surgery for analysis. The patients were matched in terms of age, chemotherapy, molecular breast cancer subtype (Her2 subtype, luminal, and triple‐negative subtypes based on immunohistochemistry abbreviation), lymph node status and site (recruited at Stavanger or Haukeland University Hospitals). Next‐generation sequencing of tissue from the primary tumors (*n* = 70) and the systemic relapses (if available (*n* = 21/35)) was performed retrospectively for all patients. A total of 230 plasma samples, from the 70 breast cancer patients, were assessed for ctDNA. In addition, we analyzed blood samples obtained from 35 age‐matched healthy women.

**Fig. 1:**
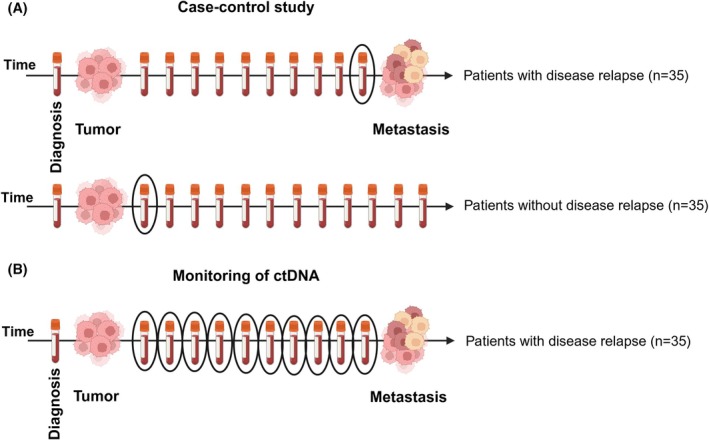
Study design. (A) We first assessed the circulating tumor DNA (ctDNA) level in plasma samples obtained at the time of relapse from 35 breast cancer patients who experienced disease recurrence and compared it to the ctDNA level in the first plasma sample obtained after surgery in matched patients without disease recurrence for the first 5 years of follow‐up. (B) We then retrospectively analyzed plasma samples obtained every 6–12 months from the 35 patients with disease recurrence (circled tubes) in order to monitor the ctDNA level during follow‐up for the detection of minimal residual disease at an early time point (Created in BioRender. Nordgård, O. (2025) https://BioRender.com/x53s049).

From the patients experiencing disease relapse, all plasma samples (*n* = 196) obtained between the time of inclusion and metastatic disease confirmed by radiologic imaging, were analyzed. For comparison, we also analyzed the first plasma sample (*n* = 35) obtained up to 3.5 years after surgery (range 0–42 months) from matched patients without disease relapse during the first 5 years of follow‐up. The study methodologies conformed to the standards set by the Declaration of Helsinki, and the study design is shown in Fig. 1.

The project was approved by the Regional Committee for Medical and Health Research Ethics ((ID #2010/1957) and (ID #2015/2010)), and the study was performed in compliance with the ethical regulations. Written informed consent was obtained from all participants.

### 2.2 DNA isolation from plasma and whole blood samples

All blood samples were collected in EDTA tubes, and plasma was isolated either by centrifugation for 10 min at 2200 ***g*** and room temperature or by density gradient centrifugation according to the protocol of the manufacturer (Lymphoprep; Axis‐Shield, Serumwerk Bernburg, Bernburg, Germany). The blood samples were processed at room temperature, and the processing time varied from 15 to 45 min. The plasma samples were then stored on ice before aliquotation, and long‐term storage at −80 °C until further use. Circulating cell‐free DNA (cfDNA) was isolated from 1 mL undiluted plasma or 4 mL density gradient plasma using the ‘QIAamp circulating nucleic acid kit’ (Qiagen, Hilden, Germany), according to the manufacturer's procedure, and all samples were eluted in 50 μL AVE buffer. In addition, genomic DNA was isolated from whole blood samples (1.5 mL), for ctDNA‐positive patients, to identify clonal hematopoiesis of indeterminate potential (CHIP). We also analyzed blood samples obtained from three healthy controls in whom we detected several variants in plasma, to check if these variants were due to CHIP. The genomic DNA was isolated using the ‘QIAamp DNA blood Midi kit’ (Qiagen) and eluted in 50 μL AE buffer. All the DNA/cfDNA concentrations were assessed by the ‘Qubit 1× dsDNA HS Assay kit’ (Invitrogen, Carlsbad, CA, USA) using the Qubit 2.0 Fluorometer (Thermo Fisher Scientific, Waltham, MA, USA).

### 2.3 DNA isolation from tissue samples

Tissue samples were processed and stained according to previously published protocols [23]. Samples were fixed in 10% neutral buffered formalin, dehydrated, and embedded in paraffin. Formalin fixed, paraffin embedded (FFPE) sections of 4 μm were stained on Superfrost Plus^®^ slides with Hematoxylin and Eosin. Slides were assessed for tumor content (TC) by an experienced breast pathologist. DNA from tissue sections of 10 μm was extracted using the ‘Allprep DNA/RNA Mini kit’ (Cat. No 80204; Qiagen) following manufacturers' procedures. Median TC was 60% (range 5–95%), with 59% of samples having TC percent > 50.

### 2.4 Library preparation and sequencing of DNA from FFPE primary tumors and metastases

Libraries for DNA sequencing of tissue samples were prepared with 10 ng DNA input from each sample, using the Oncomine Comprehensive Assay v3C (cat. no. A35806; Thermo Fisher Scientific) on an Ion Chef automated library preparation system. This sequencing assay enables detection of somatic single‐nucleotide variants (SNVs), insertions and deletions (INDELs) across 161 genes of which 87 are hotspot genes, and 43 copy number variations (CNVs), with full exon coverage of 48 genes (Table S1). Eight samples were pooled in combined libraries to be sequenced on Ion 540 chips. Templating was performed by an automated Ion Chef system with the Ion 540™ Kit‐Chef (2 sequencing runs per initialization) (Cat. no A30011; Thermo Fisher Scientific) after diluting the final library to 50pM. Finally, the templated libraries were sequenced by an Ion GeneStudio S5™ sequencer following the manufacturers' recommendations, obtaining a median (range) target coverage of 2335× (415×–140 000×).

### 2.5 Library preparation and sequencing of plasma cfDNA

To detect ctDNA, cfDNA sequencing libraries were prepared with a median cfDNA input of 8.8 ng from each patient sample using the Oncomine™ Breast cfDNA Research Assay v2 (Cat. no A35865; Thermo Fisher Scientific). This assay can detect SNVs and CNVs in 12 frequently mutated breast cancer genes (152 hotspots in *AKT1, EGFR, ERBB2, ERBB3, ESR1, FBXW7, KRAS, PIK3CA, SF3B1, TP53, CCND1*, and *FGFR1*) (Table S2). In brief, the cfDNA fragments were marked with molecular barcodes, in addition to sample barcodes, during two consecutive rounds of PCR amplification to enable discrimination between true and false SNVs for the detection of ctDNA‐positive samples. Following amplification, all libraries were cleaned two times with Agencourt™ AMPure™ XP beads (Cat. no. A63880; Beckman Coulter, Brea, CA, USA) before the libraries' quantity and quality were assessed with the High Sensitivity DNA kit (Cat. No. 5067‐4626; Agilent, Santa Clara, CA, USA) on a 2100 BioAnalyzer instrument (Agilent). The final libraries were then diluted to 100 pm, and 10 libraries were pooled for sequencing (in each run) before downstream template preparation.

The first step in template preparation for sequencing ctDNA was to perform emulsion PCR (emPCR). During this step, the libraries are coupled to Ion Spheres particles (ISPs) before amplification is performed in oil droplets on an Ion OneTouch™ 2 Instrument, using the Ion PI™ Hi‐Q™ OT2 Reagents 200 template kit (Cat. no. A26434; Thermo Fisher Scientific). Following emPCR, the number of ISPs with libraries was measured using the Ion Sphere™ Quality Control kit (Cat. no. 44686656; Thermo Fisher Scientific), where a measurement of 10–25% templated ISPs indicates an optimal templated library allowing us to proceed to sequencing. The sequencing products were then purified on the Ion ES unit to remove unbound ISP particles, before next‐generation sequencing was performed on the Ion Proton sequencer using the Ion PI™ v3 chip (Cat. no. A26771; Thermo Fisher Scientific) and the Ion PI™ Hi‐Q™ Sequencing 200 kits (Cat. no. A264; Thermo Fisher Scientific) with a median molecular coverage of 1389×.

### 2.6 Variant calling

The raw sequencing data were automatically processed by the Ion Torrent Suite Software (version 5.12.0 and 5.18.1) before being uploaded to the Ion Reporter Software version 5.18.2.1 for variant detection. cfDNA variants were called using the Oncomine TagSeq Breast v2 Liquid Biopsy—w2.4 workflow with a modified Oncomine Extended filter chain requiring a variant allele frequency (VAF) ≥ 0.3%, at least 3 molecular reads supporting the variant, and localization outside homopolymers longer than 5 bases. On the contrary, silent mutations were not excluded nor were known polymorphisms (5000Exomes, ExAC, and UCSC Common SNPs databases) present at VAF < 30% (and thus less likely to be germline variants). However, if a variant was detected both in white blood cells and plasma from the same patient, it was categorized as a variant caused by CHIP. Additionally, if a variant was present in plasma samples from the healthy control group, the patient sample had to have a higher VAF for this variant to be categorized as a ctDNA‐positive sample. From the analysis of plasma samples from the 35 healthy women, a total of 26 variants were detected in 9 of the women in the genes *TP53* (21 variants), *PIK3CA* (4 variants), and *ERBB3* (1 variant). The VAF ranged from 0.27% to 3.43%. A patient plasma sample was considered ctDNA positive if a variant passed all the criteria above.

DNA variants in FFPE tissue biopsies were called using the Oncomine Comprehensive v3 w4.2 DNA Single Sample workflow with a custom filter requiring a VAF > = 5%, coverage > = 400, and inclusion in the Oncomine™ annotation filter. For samples with a deamination score > 100, a limit of 10% VAF was applied (*n* = 9 samples). Copy number aberrations were included if the copy number exceeded 6 copies for TC > 20%, 10 copies for 10% < TC < 20%, and 15 copies for TC < 10%. In addition, if a ctDNA variant was called in a patient's plasma sample, that variant was included even if it did not pass the Oncomine™ filter.

### 2.7 Statistical analyses

The statistical analyses were performed by SPSS version 29.0.0 and by R v.4.2.3 and v4.4.0 using the R packages maftools v2.18.0 and swimplot 1.2.0. The primary endpoint was to investigate whether there were differences in ctDNA positivity between patients in the case and control groups, while the secondary endpoint was to assess how long before the relapse a positive ctDNA test could be detected. Both intention‐to‐monitor and tumor‐informed sensitivities (number of ctDNA‐positive cases divided by the number of patients with relapse/number of patients with relapse that had tumor mutations in the genes covered by the cfDNA panel) were determined. The blood sample obtained nearest to the date of systemic recurrence was considered the blood sample obtained at the time of relapse for each patient with disease recurrence (case). We compared the number of patients (cases) that had a ctDNA‐positive sample at the time of relapse to the number of ctDNA‐positive samples among the 35 matched patients without relapse (controls) using Fisher's exact test. 95% confidence intervals were estimated using nonparametric bootstrapping (1000 resamples) applied to contingency tables. The median concentration of ctDNA, the median amount of input cfDNA (ng), and the median molecular coverage in these two groups were also assessed by the Mann–Whitney *U*‐test. If the plasma sample at the time of relapse was ctDNA positive, we continued mutation tracking back in time to investigate how early we could detect systemic relapse. ctDNA detection in a sample was considered as a relapse detection if it was not followed by a subsequent ctDNA‐negative sample. Only samples collected after surgery were considered for relapse detection. Lead times were computed as the time differences between the date of ctDNA‐based relapse detection and the date of radiological relapse detection. For patients with biopsy‐verified systemic relapse, the date of biopsy was used as the date of radiological relapse detection. In the tissue biopsy analysis, mutual exclusivity of genes and clinical enrichment analysis were analyzed using pairwise Fisher's exact tests. Where multiple comparisons were made, *P*‐values were corrected using the Benjamini–Hochberg false discovery rate correction. Illustrations of the primary tumor and metastasis were made in BioRender.

## 3 Results

### 3.1 ctDNA detection in operable breast cancer patients

**Fig. 2:**
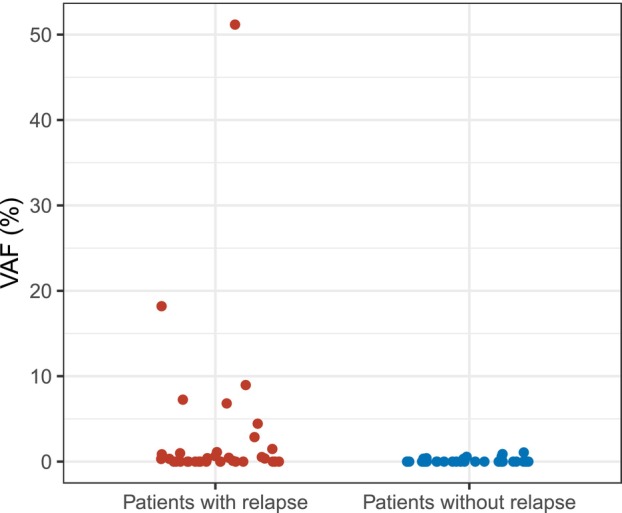
Variant allele frequency (%) of tumor variants in plasma cfDNA from patients with and without relapse. For patients with relapse (cases; *n* = 35), a sample obtained at the time of relapse was analyzed, whereas the first sample obtained after surgery was analyzed for patients without relapse (controls; *n* = 35).

In this case–control study, 35 patients with primary operable breast cancer and later systemic disease relapse were examined for the presence of ctDNA in peripheral blood samples obtained at the time of relapse. Blood samples obtained from 35 matched patients with operable breast cancer, but without known later disease relapse, were also examined in comparison. The clinicopathological characteristics of the cases (patients with relapse) and controls (patients without relapse) were generally similar (Table 1), and however, the patients with relapse had a higher T‐stage compared to the patients without relapse (*P* = 0.016). Among the 35 patients with relapse, 19 (54%; 95% CI: 37–71%) patients had ctDNA detected at the time of systemic relapse. For one of these patients, the blood sample obtained at the time of disease relapse was obtained before surgery because of a short time to relapse. Among the 35 matched patients without disease relapse, the first plasma sample obtained after the breast cancer surgery was ctDNA positive in seven (20%) patients. A significantly higher number of patients with relapse were ctDNA positive at the time of relapse compared to the matched patients without relapse (*P* = 0.006). The median concentration of ctDNA measured in the samples from patients with disease relapse was also significantly higher than the concentrations measured in the samples from patients without relapse (*P* = 0.002, Fig. 2). Moreover, the median amount of input cfDNA was higher for the patients with relapse (*P* = 0.026), while the median molecular coverage did not differ significantly between patients with and without disease relapse. Comparing ctDNA‐positive and ctDNA‐negative patients with disease relapse revealed no difference in cfDNA input amount between the groups; however, there was a significant difference in median molecular coverage (*P* = 0.045). Furthermore, no difference was observed between ctDNA‐positive and ctDNA‐negative patients with disease relapse regarding clinical characteristics and metastatic sites, nor among patients without relapse.

**Table 1:** Clinical characteristics of the patient cohort. Calculations were performed using the chi‐square test.

Variables	All patients	Patients with relapse	Patients without relapse	*P*‐value
	*N* = 70	*N* = 35	*N* = 35	
**Age**				
< 55 years	27	13	14	1.00
≥ 55 years	43	22	21	
**Histological type (%)**				
Lobular	5	2	3	0.106
Ductal	56	25	31	
Other/unknown	9	8	1	
**Tumor grade (%)**				
1	13	7	6	0.489
2	14	5	9	
3	43	23	20	
**Tumor size (%)**				
pT1	37	13	24	**0.016**
pT2–4	33	22	11	
**Lymph node status (%)**				
pN0	35	15	20	0.339
pN1–3	35	20	15	
**Estrogen receptor status (%)**				
ER positive	47	22	25	0.611
ER negative	23	13	10	
**Progesterone receptor status (%)**				
PgR positive	33	13	20	0.15
PgR negative	37	22	15	
**HER2 status (%)**				
Positive	19	9	10	0.500
Negative	51	26	25	
**Adjuvant therapy (%)**				
Chemotherapy	60	30	30	1.00
Endocrine therapy	44	21	23	0.805
HER2 treatment	19	9	10	1.00

Bold values indicate a significant difference between groups.

### 3.2 Detection of systemic relapse by ctDNA monitoring

**Fig. 3:**
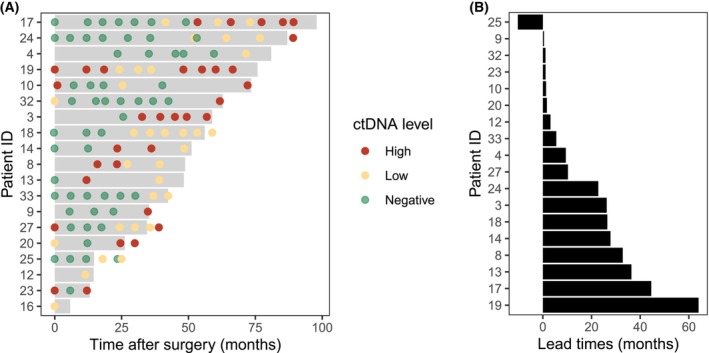
ctDNA monitoring in patients with ctDNA detected at disease relapse. (A) Swimmer plot showing time from surgery to systemic relapse (gray bars) and ctDNA measurements over time (colored circles) for individual patients (ID numbers shown in *y* axis). The color of the circles shows the ctDNA level (green: negative, yellow: low (0 > and ≤ 1% VAF), red: high (> 1% VAF)), whereas the sampling time points range from before surgery (at the time of inclusion) until 1 year after systemic disease relapse. (B) Lead times between ctDNA‐based and radiological detection of disease relapse for individual patients (ID numbers shown in *y* axis). Only blood samples collected after surgery were used to determine lead times.

All plasma samples collected from patients with ctDNA detected at the time of disease relapse (*n* = 19) were subsequently analyzed for the presence of ctDNA in samples collected at earlier timepoints to estimate lead time of relapse detection by ctDNA (Fig. 3A). In 17 (89%) of these patients, ctDNA detection after surgery indicated disease relapse earlier than radiological examination (*P* = 0.0002), with a median lead time of 10.3 months (95% CI 1.64–27.7 months; IQR 1.64–27.8; Fig. 3B). One patient had ctDNA‐based relapse detected 10.3 months after radiological detection. Another patient did not have any samples collected after surgery, because of a short time to systemic relapse, and was not included in the lead time analysis.

**Fig. 4:**
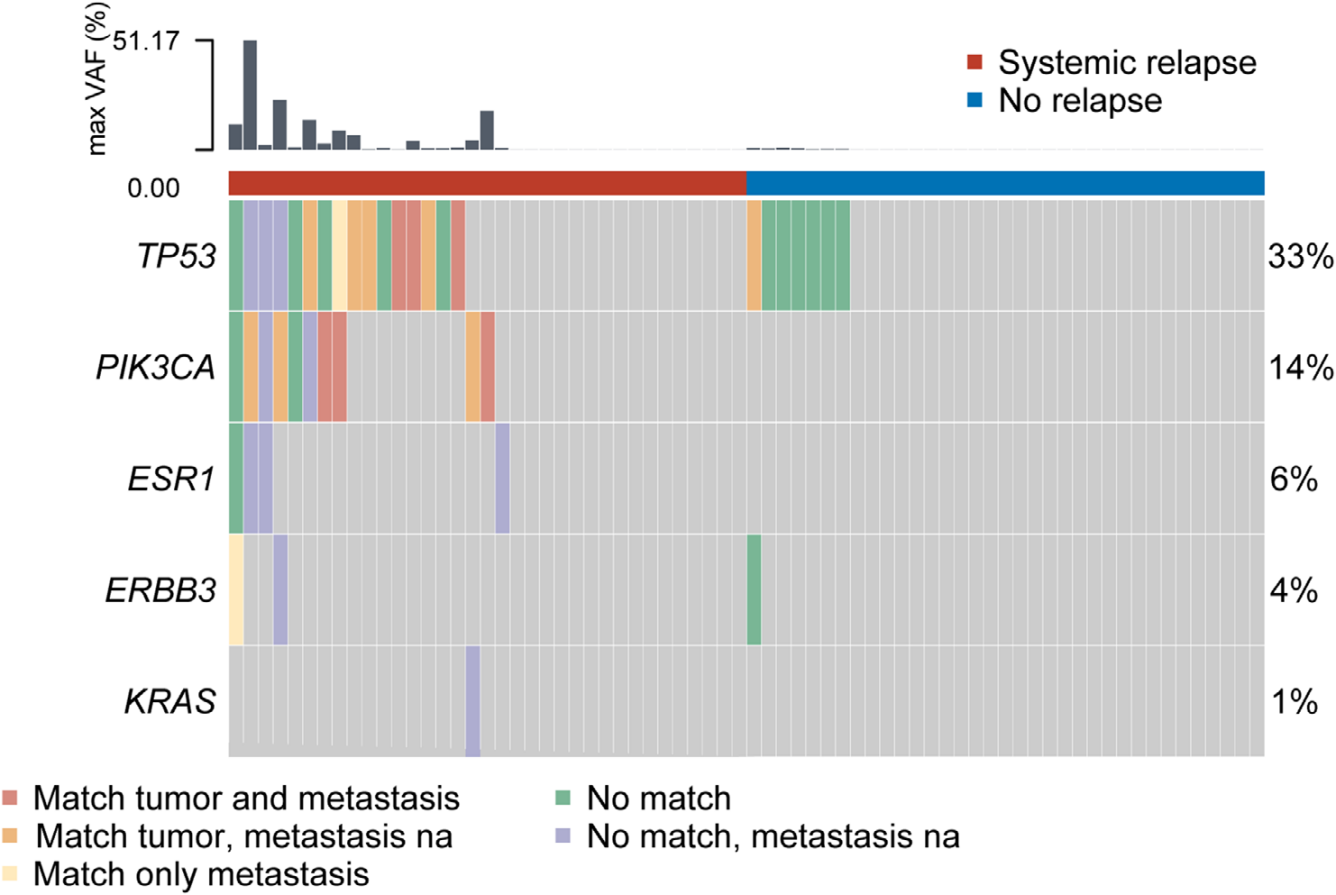
Oncoplot of ctDNA variants from patients with and without disease relapse. In this plot each patient is represented by a column, with colors representing ctDNA variants from any timepoint found in either only tumor (orange), only metastasis (yellow), both tumor and metastasis (red), or no match to tumor or metastasis (green or if no available metastasis, purple). The rows correspond to genes with detected mutations. One tile may represent several variants in the same gene. The columns are arranged by patients with (left/red bar) and without relapse (right/blue bar) and then by variant profile. The top bar shows the maximum variant allele frequency (VAF; percentages) for each patient. The numbers at the right show the percentages of patients with ctDNA mutations detected in each gene.

In the analyzed plasma samples, a total of 271 genetic variants were detected that were not CHIP variants (Fig. 4). Of these 271 variants, there were 208 unique patient variants, excluding variants detected multiple times per patient. The most frequently mutated genes were *TP53* with 209 variants (77%), followed by *PIK3CA* with 41 variants (15%), *ESR1* with 15 variants (5.5%), *ERBB3* with 5variants, (1.8%), and *KRAS* with 1 variant (0.3%). In addition, 66 variants were also found in white blood cells and excluded as being CHIP variants.

### 3.3 Genomic characterization of primary tumors and metastases

**Fig. 5:**
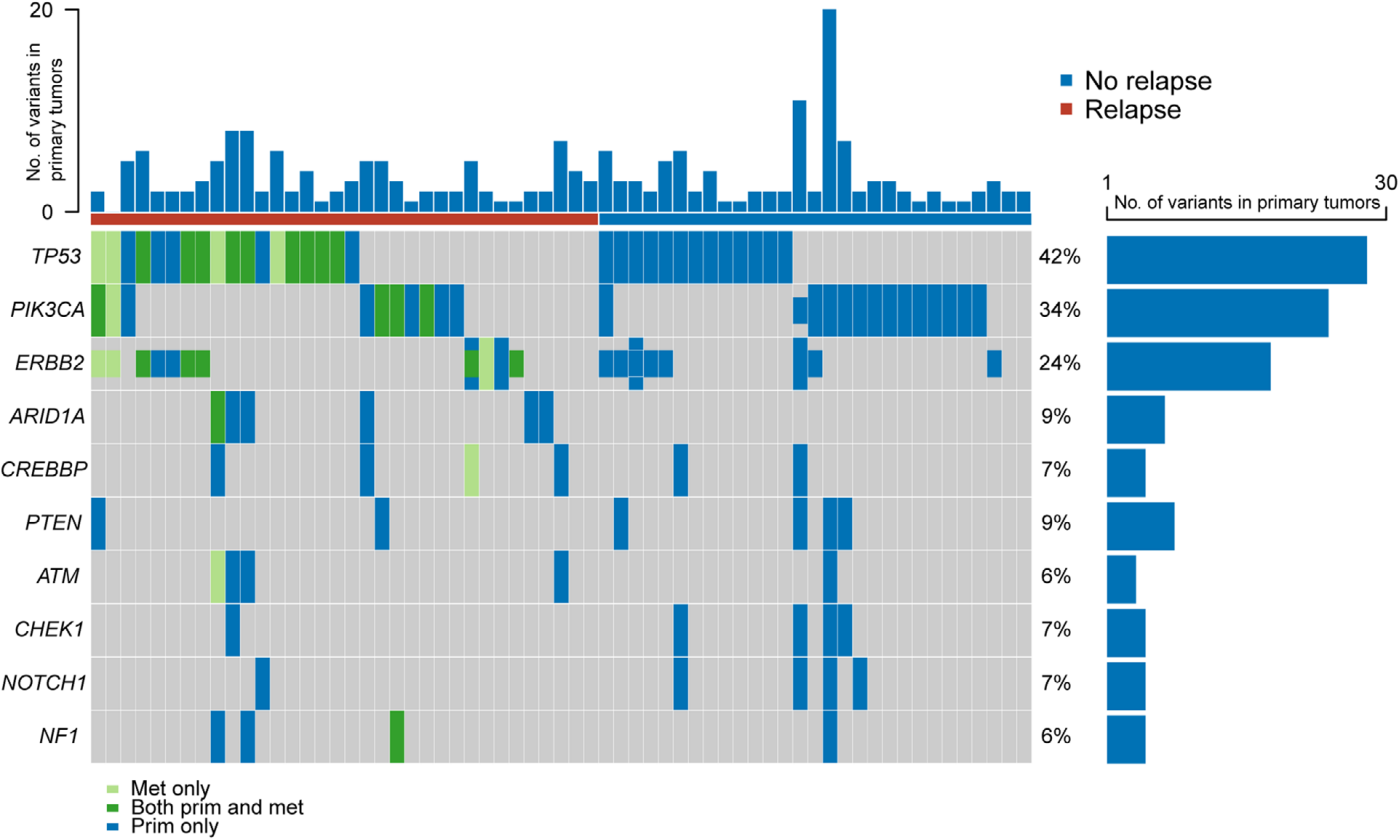
Oncoplot of genetic alterations in tissue biopsies from patients with and without relapse. Every row represents one gene, with colors designating whether alteration is present in tumor, metastasis, or both. Amplifications are represented by short bars. Only the ten most commonly altered genes are shown. Each column represents one patient. The columns are arranged by patient status with (left/red bar) and without relapse (right/blue bar) and then by variant profile. The top and side bar plots represent the number of variants found in each primary tumor biopsy and gene, and the numbers on the right represent the percentage of biopsies in which alterations are found. Each tile may represent several alterations. Oncoplot is based on 97 primary tumor or metastasis samples from 67 patients, of which six patients had no detected alterations (not plotted). Abbreviations: prim, primary tumor biopsy; met, metastasis biopsy.

In this case–control study, we also analyzed tumor tissue biopsies from included patients to identify tumor mutations expected to be found in plasma and to characterize the concordance between tissue biopsies and plasma cfDNA regarding genetic changes. For patients experiencing disease relapse, we also sequenced tissue biopsies from all available metastases. In total, 99 tissue samples from 69 of the included patients, including one or more metastasis tissue samples from 22 patients, were analyzed by targeted DNA sequencing. One primary tumor was unavailable and two samples from primary tumors did not reach the criteria for quality control, leaving 97 samples from 67 patients available for analysis. A total of 153 variants were found in the primary tumors, with 15 additional variants in samples from the metastases (Fig. 5). There was no difference in the number of alterations (median 1, range 0–20) between patients with and without disease relapse (*P* = 0.42). The most frequently altered genes in primary tumors were *TP53* (28 variants, 42% of patients), *PIK3CA* (2 variants, 34% of patients), and *ERBB2* (16 variants, 24% of patients). Most frequent novel mutations in metastasis samples were found in the *TP53* (4 variants) and *EGFR* (2 variants) genes. Novel *ERBB2* amplifications in metastases were found in two patients. Among the patients with both tumor and metastasis samples available (*n* = 22), 10 patients had mutations unique to the metastasis sample. *TP53* and *PIK3CA* variants were mutually exclusive (*P* = 0.001) (Fig. S1). *TP53* was more often mutated in ER‐negative patients (*P* = 0.007).

### 3.4 Concordance between tissue and ctDNA findings

We examined the concordance between the genetic variants found in plasma cfDNA and the biopsies from primary tumors and metastases from all the ctDNA‐positive patients. As the target regions of the panels used for ctDNA and tissue profiling did not fully overlap, we only focused on variants in genes covered by both panels. In the ctDNA‐positive patients with relapse, there was concordance between ctDNA variants in any sample and primary tumor variants in 13 of 19 patients (68%). Six of these patients did also have metastasis with the same variant. ctDNA variants unique to the metastasis were detected in two patients with relapse (Fig. 4). In total, 16 ctDNA variants were detected in biopsy samples from the metastases (Fig. 4). In the matched patients without disease relapse, only one of the detected ctDNA variants was found in primary tumors (one patient was missing primary tumor data).

**Fig. 6:**
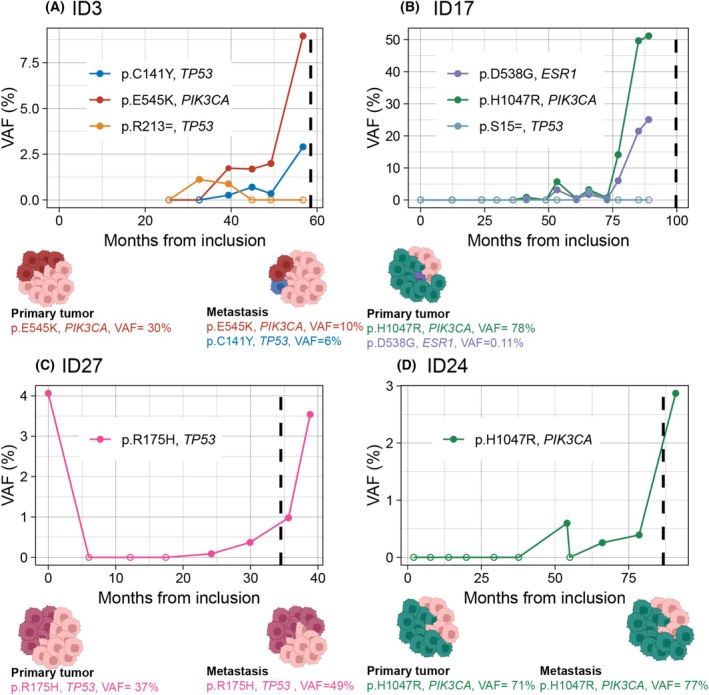
Monitoring of ctDNA levels in four patients developing systemic relapse. Monitoring of four individual patients (A–D). Variant allele frequencies (VAF) of specific detected genomic variants are shown as a function of time after inclusion in the study. Time of systemic relapse based on radiological evidence is shown as a dashed vertical line for each patient. Empty dots indicate that the variant was not detected at this timepoint. Under each plot, an illustration of the same variants found in primary tumor and metastasis tissue is shown (created in BioRender).

Looking in depth at selected patients with disease relapse, we observed that the ctDNA analyses appeared to reflect the total tumor load in the patients (Fig. 6A–D). In most cases, the ctDNA variants were also present in the primary tumor. However, we also found a case where a ctDNA variant was unique to the metastasis (Fig. 6A). In one case without available metastasis tissue, a ctDNA variant with a very high‐plasma VAF was detected, while displaying a low VAF in the primary tumor, probably reflecting a dominant clone in the metastasis (Fig. 6B).

Among the 14 (37%) ctDNA‐negative patients with relapse, two patients had no variants covered by the Oncomine Comprehensive Assay v3C in their primary tumors, reducing the likelihood of ctDNA detection with our target panel in these patients. In addition, there were two patients with primary tumor variants only in genes that were not covered by the ctDNA panel. Hence, only 31 of the 35 patients with recurrence had primary tumor variants that could be detected in cfDNA, resulting in an adjusted ctDNA detection rate of 61% (19 of 31; 95% CI: 45–78%). Accordingly, in the remaining 11 ctDNA‐negative cases, ctDNA assessment failed despite the presence of tumor variants.

## 4 Discussion

In this study, we demonstrate early noninvasive detection of metastatic disease by monitoring ctDNA. As the results show, systemic relapse is detected months prior to radiologically confirmed relapse in a significant proportion of patients. In our study, 19 of 35 (54%) patients with systemic disease were ctDNA positive at the time of relapse. In 17 patients, ctDNA detection revealed the relapse prior to radiological confirmation. The median follow‐up time in our study was 99 months, and the median lead time was 10.3 months.

ctDNA in breast cancer patients after curative treatment has been investigated in several studies [7, 16, 18, 20, 24, 25]. In a comprehensive study by Shaw *et al*. [7], ctDNA was detected prior to clinical or radiologic relapse in 30 out of 34 patients (88.2%). The median follow‐up time was 77 months, and the median lead time was 10.5 months. Similarly, Olsson and colleagues detected ctDNA in 12 of 14 patients (86%) with a median lead time of 11 months [18]. In our study, the detection rate was somewhat lower, as 19 of 31 patients (61%, four did not have primary tumor mutation in the target genes) had ctDNA detected at the time of disease recurrence. Several factors may explain this discrepancy. In all the studies mentioned above, ctDNA detection was based on tumor‐informed mutations found by whole‐exome sequencing of the primary tumors, which provides increased sensitivity. Shaw *et al*. [7] used a tumor‐informed targeted sequencing‐based ctDNA assay, developed from whole‐exome sequencing data from the primary tumor, targeting 16 high‐ranked, clonal somatic single‐nucleotide variants for each patient. In contrast, we used a smaller tumor‐independent panel, the Oncomine™ Breast cfDNA Research Assay v2, which includes 10 genes and 80% of the coding exons of *TP53*. The size of our panel may have contributed to a lower detection rate, as exemplified by two patients for whom we did not detect any variants in either the primary tumors or in plasma. Another factor contributing to the lower detection rate in our study compared to the study by Shaw *et al*. may be that Shaw *et al*. define patients with relapse as ctDNA positive if a single positive sample is detected at any time point following surgery. This definition is slightly less rigid compared to ours, as we define patients with relapse as ctDNA positive only if we detect ctDNA in the last sample obtained at the time of the radiologically confirmed relapse. Hence, because we initially only analyzed one sample per patient, the likelihood of a ctDNA finding is lower. Technical challenges could also have been an explanation for the lower ctDNA detection rate; however, this did not appear to be the case in our study. For instance, among our patients with relapse, no significant difference was observed in cfDNA input between patients with detected ctDNA and patients without. Moreover, there was significantly higher median molecular coverage of the DNA sequencing panel in the ctDNA‐negative patient group (*P* = 0.045). Thus, lower input amount and sequencing depth could not account for the absence of ctDNA detection in the ctDNA negative group *per se*. Nevertheless, higher cfDNA input and the use of a larger cfDNA panel could have further increased the sensitivity [26].

We observed a median lead time of 10.3 months between ctDNA‐ and radiology‐based detection of recurrence. This number is comparable to those reported previously in similar studies of patients with recurring operable breast cancer [7, 20], a minor increase in ctDNA levels in the subgroup of patients where we detect ctDNA at the time of relapse but totally fails to detect ctDNA in other patients. One explanation for this could be our limited gene panel, not covering all tumor variants in the patients, as discussed above. Another explanation could be differences between the patient cohorts in terms of ctDNA shedding. Our cohort closely resembles the cohort described in Shaw *et al*. in terms of molecular subtypes. However, they selected high‐risk patients based on an online relapse score of over 65% or a 10‐year mortality score over 50% [7]. An interesting observation is that Shaw *et al*. found that all four patients without detectable ctDNA were HR+/HER2‐, three of whom had bone metastases, and one malignant pleural effusion without other metastases. Milder phenotypes, prolonged treatment durations, and relapse sites such as bone, brain, or local progression, along with HR+ and HER2‐ disease, apparently posed greater challenges for ctDNA detection compared to other breast cancer subtypes [7], while high levels of ctDNA were particularly observed in triple‐negative breast cancer [7, 20]. In our cohort, we found that 11 patients with disease relapse were negative for ctDNA despite having target panel mutations present in their primary tumors. We therefore tested for an association between ctDNA detection and clinical characteristics, as well as dissemination patterns to metastatic sites, but found no significant associations in our study.

Even though detection of increased ctDNA levels can reveal breast cancer recurrence before it is confirmed radiologically, the therapeutic benefit for the patients still remains unclear [26]. The ultimate goal of early relapse detection is to improve patient outcome by earlier therapeutic treatment. However, although detection of molecular relapse may precede clinical recurrence by many months, this lead time does not inherently demonstrate a survival benefit. Several ongoing trials are currently testing whether early intervention guided by MRD positivity, mutation tracking or patient‐level data can improve outcomes. The SERENA‐6 trial has for instance shown that the switch of palliative treatment at the time of molecular detection of resistance mutations prolongs progression‐free survival (PFS) and improves quality of life [27]. Overall survival data are not yet mature, but it will be crucial in determining the ultimate benefit of ctDNA‐guided treatment adjustment in the advanced setting. In contrast, results from the c‐TRAK TN trial, where they have investigated whether ctDNA‐based intervention improves survival in breast cancer patients in a neoadjuvant setting, were unfortunately inconclusive [25]. Thus, larger prospective intervention studies based on tailored follow‐up are needed to further investigate if detection of minimal residual disease can improve patient survival, especially in the adjuvant setting where the disease burden is lower. Until the clinical utility of early intervention is established, the implementation of ctDNA diagnostics in routine clinical practice remains premature as patients diagnosed with minimal residual disease risk reduced quality of life and psychological distress without knowing whether they have improved outcomes.

Regarding ctDNA detection in patients without disease relapse, we detected ctDNA, in the first plasma sample obtained after surgery, in 7 of 35 patients. A plausible explanation for this is that these seven patients may have had minimal residual disease without experiencing clinical relapse during the five‐year follow‐up period, which aligns with our understanding of late recurrence [28]. The low levels of ctDNA detected in these seven patients (Fig. 2) further support the idea of minimal disease at the sampling timepoint, in contrast to the levels observed at the time of radiologically confirmed systemic disease. Several studies have reported that occasional detection of ctDNA following surgical treatment is associated with an increased risk of recurrence [20, 29, 30]. Alternatively, the results may indicate the presence of another malignancy or mutated hematopoietic cell clones without clear clinical relevance. It is well established that CHIP is more common with increasing age [20, 31], and such samples could potentially be misinterpreted as ctDNA positive [20, 32]. We attempted to account for CHIP by isolating genomic DNA from whole blood samples for all ctDNA‐positive patients to minimize the number of false‐positive results. However, it is possible that not all cases were identified. Moreover, among patients without recurrence no significant differences in cfDNA input, or molecular coverage were observed between those with and without ctDNA findings. Due to our study design, we cannot assess the temporal variations in ctDNA level in the control group, and further research is needed to fully understand the significance of occasional ctDNA positivity in patients without disease recurrence.

By sequencing both primary tumors and metastasis in this study, we were able to examine tumor cell heterogeneity in relation to ctDNA detection. In this context, 16 variants were detected in both cfDNA and available biopsies of patients with relapse, whereas 51 variants were found exclusively in the biopsies. Figure 6 illustrates ctDNA levels in four patients who developed systemic relapse, with patient ID3 and ID17 demonstrating cancer cell heterogeneity. In ID3, ctDNA reveals two new genomic variants in *TP53* that were not found in the primary tumor, in addition to increased levels of the *PIK3CA* variant, which was detected in the primary tumor. In ID17, variants in *ESR1* and *TP53* were detected, along with increased levels of the *PIK3CA* variant, which was known from the primary tumor. These examples illustrate how ctDNA can provide valuable Supporting Information to conventional tissue sampling and may serve as a substitute for tissue biopsies in cases in which obtaining a conventional biopsy is challenging [33]. ctDNA has the potential to reflect the entire tumor burden of the patient, thereby providing insights into potential targeted treatments that single biopsies of the primary tumor may not reveal. However, since many tumor variants were not detected in cfDNA in our study, further research is warranted before concluding whether ctDNA testing can serve as a substitute to tissue biopsy in operable breast cancer patients. In breast cancer in particular, treatment of metastatic patients is largely guided based on ER/PgR and HER2 status. Thus, for ctDNA analysis to replace biopsy of metastases, these molecular markers must also be covered by the ctDNA assay, which is not the case for ER/PgR with our current assay. However, although using larger gene panels ctDNA cannot be detected in all patients, even with metastatic cancer [7, 34, 35]. Thus, adding other comprehensive molecular assessments of blood samples, like large‐scale RNA, protein and metabolite measurements, and even their computational integration, may contribute to increase the clinical usefulness of blood‐based cancer diagnostics.

## 5 Conclusion

Our data demonstrate that ctDNA monitoring can reveal disease relapse earlier than radiology in a subset of patients. In 17 of 35 relapsing patients (49%), ctDNA became positive at a median of 9.9 months (range 0–64) before radiologic confirmation. The failure to detect ctDNA in some patients with recurrent disease suggests that ctDNA could serve as a supplement to other monitoring approaches. The ctDNA detected in the control group without disease relapse requires further clarification, either through longer patient follow‐up or further experimental validation. Nevertheless, larger clinical studies are needed where ctDNA is integrated into the decision‐making process to determine whether earlier treatment of metastatic disease based on ctDNA monitoring results in a survival benefit for patients.

## Conflict of interest

The authors declare no conflict of interest.

## Author contributions

EAMJ, ON, KT, MA, KJ, and BG conceptualized the study; KT, MA, and MLA performed the NGS; GM, THL, YHV, AH, KV, KL, KBH, NEA, FME, EL, and ARA were involved in patient recruitment; MA, MLA, YHV, SO, TH, KT, ACK, EG, and STS collected the tissue and plasma specimens; KT, MA, ON, KLAa, EAMJ, and JTK carried out the data analysis; KLAa, KT, MA, and ON led the writing of the manuscript; BG, KT, ON, EAMJ, MA, GM, THL, SO, MLA, YHV, KJ, AH, KV, ARA, ACK, TH, EG, JTK, KL, KBH, STS, NEA, FME, and EAL contributed to the writing—review and editing.

## Supporting information


**Table S1.** Oncomine Assay gene lists for FFPE tissue profiling: Oncomine Comprehensive Assay v3.
**Table S2.** Oncomine Assay gene list for ctDNA profiling: Oncomine Breast cfDNA Research Assay version 2.

**Fig. S1.** Variant allele frequency bar plot and somatic interaction analysis. TP53 and PIK3CA are mutually exclusive, while PIK3CA frequently co‐occurs together with PTEN, and the less frequently mutated genes more often occur together.

## Data Availability

Datasets, materials, and laboratory protocols are available upon request.

## Appendix 1

Members of the PBCB study group: Prof. Gunnar Mellgren, Tone Hoel Lende, Anette Heie, Kristin Viste, Anita Røyneberg Alvheim, Emiel AM Janssen, Kristin Jonsdottir, Ann Cathrine Kroksveen Thomas Helland, Einar Gudlaugsson, Kjersti Tjensvoll, Oddmund Nordgård Satu Oltedal, Bjørnar Gilje, Kristin Løge Aanestad, Jan Terje Kvaløy, Kirsten Lode, Kari Britt Hagen, Marie Austdal, Ylva H. Vignes, Siri Tungland Sola, Nina Egeland Amundsen, Finn Magnus Eliassen, Emeritus Ernst A Lien
