# Supplementary material for: Monitoring of circulating tumor DNA allows early detection of disease relapse in patients with operable breast cancer
Source: Mol Oncol. 2025 Nov 27;20(4):981–94. doi: 10.1002/1878-0261.70170 (PMC13060637; doi:10.1002/1878-0261.70170)
Supplement: Supplementary file 1 — Table S1. Oncomine Assay gene lists for FFPE tissue profiling: Oncomine Comprehensive Assay v3. Table S2. Oncomine Assay gene list for ctDNA profiling: Oncomine Breast cfDNA Research Assay version 2. [file MOL2-20-981-s001.docx]

**Monitoring of circulating tumor DNA allows early detection of disease relapse in patients with operable breast cancer**

Kristin Løge Aanestad^1*^, Marie Austdal^2^, Oddmund Nordgård^1,^, Gunnar Mellgren^3,8^, Satu Oltedal^1^, Marie L. Austbø ^4^, Ylva H. Vignes ^5^, Thomas Helland^3,8^, Kristin Jonsdottir ^2^, Tone H. Lende ^5^, PBCB study group, Emiel A.M. Janssen^4,6,7^, Bjørnar Gilje^1^, Kjersti Tjensvoll^1^.

1. Department of Hematology and Oncology, Stavanger University Hospital, N-4068 Stavanger, Norway.
2. Department of Research, Stavanger University Hospital, N-4068 Stavanger, Norway.
3. Hormone Laboratory, Department of Medical Biochemistry and Pharmacology, Haukeland University Hospital, N-5021 Bergen.
4. Department of Pathology, Stavanger University Hospital, N-4068 Stavanger, Norway.
5. Department of Surgery, Stavanger University Hospital, N-4068 Stavanger, Norway.
6. Department of Chemistry, Bioscience and Environmental Engineering, University of Stavanger, N-4021 Stavanger, Norway.
7. Institute for Biomedicine and Glycomics, Griffith University, Queensland, Australia.
8. Department of Clinical Science, University of Bergen, N-5020 Bergen, Norway.

*Corresponding author:

Kristin Løge Aanestad, Department of Hematology and Oncology, Stavanger University Hospital, N-4068 Stavanger, Norway.

E-mail: [kristin.loge.aanestad@sus.no](mailto:kristin.loge.aanestad@sus.no).

Tel: +47-90882951

**Table S1**: Oncomine Assay gene lists for FFPE tissue profiling: Oncomine Comprehensive Assay v3

| Hotspot genes (87) | Copy number variants (43) | Full exon coverage (48) |
| --- | --- | --- |
| *AKT1 AKT2 AKT3 ALK AR ARAF AXL BRAF BTK CBL CCND1 CDK4 CDK6 CHEK2 CSF1R CTNNB1 DDR2 EGFR ERBB2 ERBB3 ERBB4 ERCC2 ESR1 EZH2 FGFR1 FGFR2 FGFR3 FGFR4 FLT3 FOXL2 GATA2 GNA11 GNAQ GNAS H3F3A HIST1H3B HNF1A HRAS IDH1 IDH2 JAK1 JAK2 JAK3 KDR KIT KNSTRN KRAS MAGOH MAP2K1 MAP2K2 MAP2K4 MAPK1 MAX MDM4 MED12 MET MTOR MYC MYCN MYD88 NFE2L2 NRAS NTRK1 NTRK2 NTRK3 PDGFRA PDGFRB PIK3CA PIK3CB PPP2R1A PTPN11 RAC1 RAF1 RET RHEB RHOA ROS1 SF3B1 SMAD4 SMO SPOP SRC STAT3 TERT TOP1 U2AF1 XPO1* | *AKT1 AKT2 AKT3 ALK AR AXL BRAF CCND1 CCND2 CCND3 CCNE1 CDK2 CDK4 CDK6 EGFR ERBB2 ESR1 FGF19 FGF3 FGFR1 FGFR2 FGFR3 FGFR4 FLT3 IGF1R KIT KRAS MDM2 MDM4 MET MYC MYCL MYCN NTRK1 NTRK2 NTRK3 PDGFRA PDGFRB PIK3CA PIK3CB PPARG RICTOR TERT* | *ARID1A ATM ATR ATRX BAP1 BRCA1 BRCA2 CDK12 CDKN1B CDKN2A CDKN2B CHEK1 CREBBP FANCA FANCD2 FANCI FBXW7 MLH1 MRE11 MSH2 MSH6 NBN NF1 NF2 NOTCH1 NOTCH2 NOTCH3 PALB2 PIK3R1 PMS2 POLE PTCH1 PTEN RAD50 RAD51 RAD51C RAD51D RAD51B RB1 RNF43 SETD2 SLX4 SMARCA4 SMARCB1 STK11 TP53 TSC1 TSC2* |

**Table S2**: Oncomine Assay gene list for ctDNA profiling: Oncomine Breast cfDNA Research Assay version 2

| **Hotspot genes (12)** | **Selected SNV hotspots** | **Copy number variations (3)** | **Full exon coverage** |
| --- | --- | --- | --- |
| *AKT1 CCND1 EGFR ERBB2 ERBB3 ESR1 FBXW7 FGFR1 KRAS PIK3CA SF3B1 TP53* | >150 hotspots including:  *PIK3CA*: E545K, H1047R  *AKT1*: E17K  *ESR1*: mutations associated with anti- estrogen resistance *TP53*: mutations associated with loss of function  *ERBB2*: mutations associated with sensitivity to anti-ERBB2 therapies | *CCND1*, *ERBB2,* *FGFR1* | Expanded coverage of *TP53* |
