# Supplementary figures and images for: Monitoring of circulating tumor DNA allows early detection of disease relapse in patients with operable breast cancer
Source: Mol Oncol. 2025 Nov 27;20(4):981–94. doi: 10.1002/1878-0261.70170 (PMC13060637; doi:10.1002/1878-0261.70170)

**A**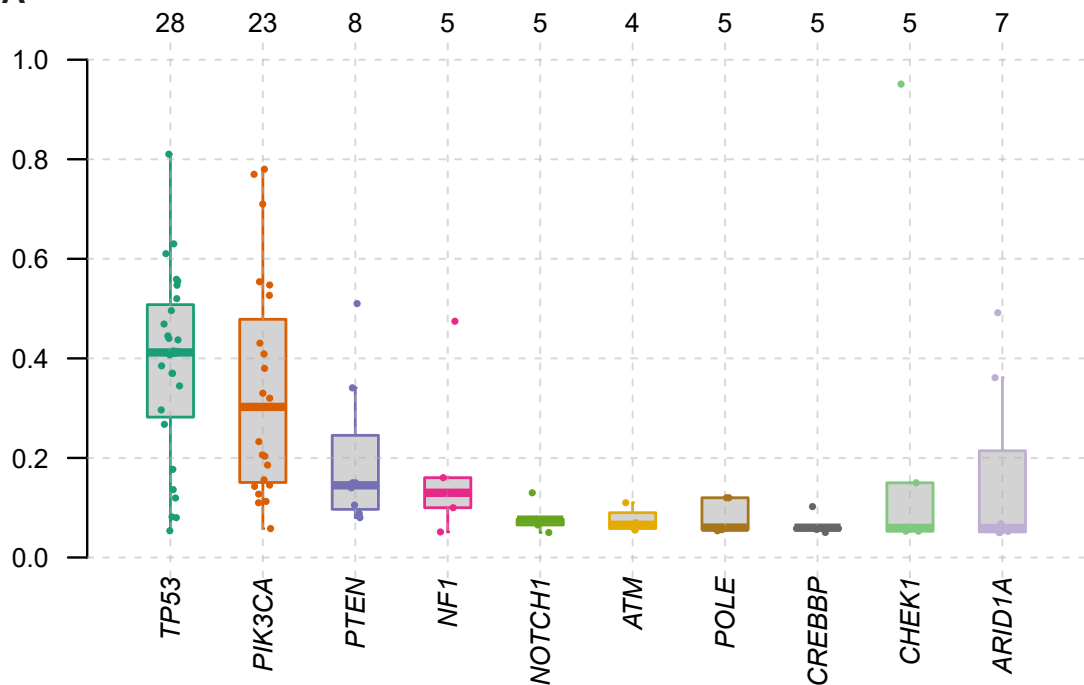**B**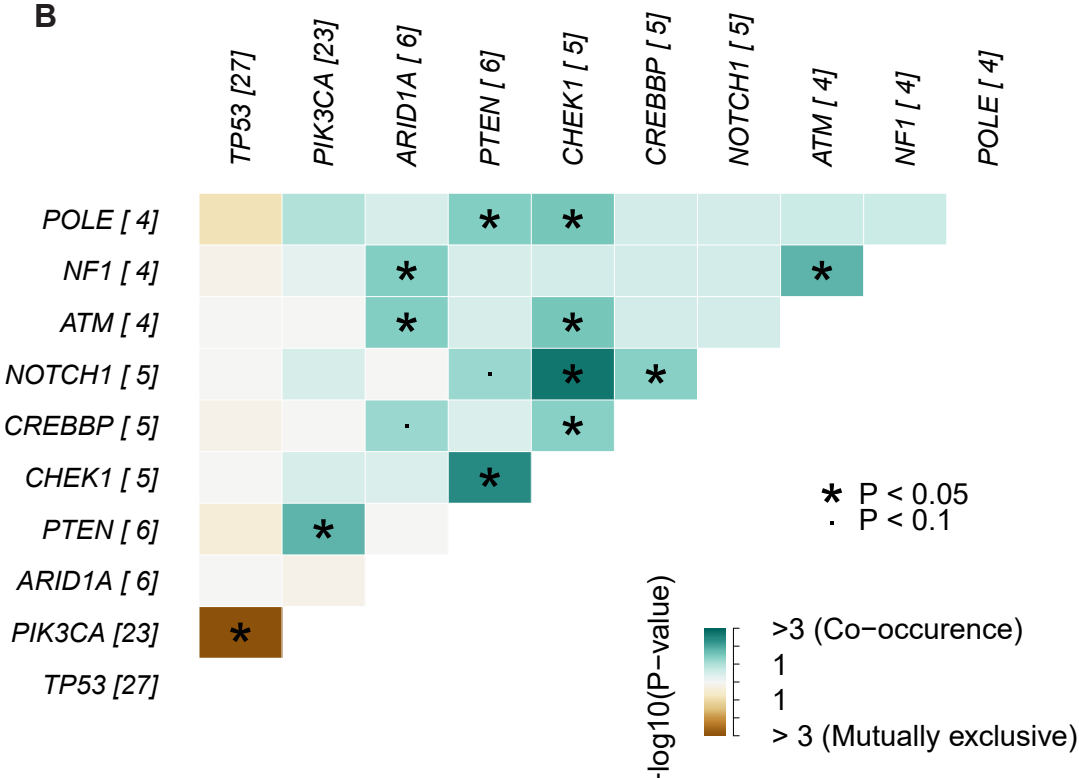

Supplement: Supplementary file 2 — Fig. S1. Variant allele frequency bar plot and somatic interaction analysis. TP53 and PIK3CA are mutually exclusive, while PIK3CA frequently co‐occurs together with PTEN, and the less frequently mutated genes more often occur together. [file MOL2-20-981-s002.pdf]
